# Supplementary material for: Ten rules for a structural bioinformatic analysis
Source: PLoS Comput Biol. 2025 Oct 31;21(10):e1013094. doi: 10.1371/journal.pcbi.1013094 (PMC12578330; doi:10.1371/journal.pcbi.1013094)
Supplement: Attachment — Submitted filename: SW_Plos Response to Reviews_update.docx [file pcbi.1013094.s001.docx]

**Comments to the Authors:
Please note here if the review is uploaded as an attachment.**

Reviewer #1: This piece is generally well written and has the potential to become a valuable guide, but it currently tries to cover too much ground and often misses the level of detail needed to be truly useful. The author should reconsider: who is the intended audience? The text swings between very basic explanations suitable for someone newly introduced to macromolecular structures and highly technical points more suited to someone capable of reprocessing crystallographic data. Narrowing the scope and defining a clear audience would significantly strengthen the piece.
There is also some "lab-speak" creeping in, terms that are familiar to experts but potentially confusing or misleading for beginners. For example, referring to coordinate files as "PDBs" may not be appropriate for those unfamiliar with the nuances of structural data/
In addition, the author should take care to represent the structure and roles of the three wwPDB partner sites (RCSB, PDBe, PDBj) more accurately. While they share a common archive, the tools, interfaces, and data visualisations each site provides are developed independently. A more neutral presentation that acknowledges the work of all three sites would be helpful. It may be worth reaching out to representatives from each to ensure the overview is current and balanced. PDBe, for instance, offers extensive visualisation tools, data aggregation features, and APIs—several of which I have noted where they could be cited but similar detail should be provided for the other sites.
In summary, this is a promising draft with a strong concept behind it, but it would benefit from:
• A clearer sense of the target audience (and consistent level of detail),
• Less reliance on insider jargon, and
• More actionable advice, including direct links to tools, training materials, and relevant publications where appropriate.

This is incredibly helpful feedback. I have updated the manuscript, aiming to narrow the language to those who are just getting into structural bioinformatics, and have asked a few colleagues to read it with feedback on where they misunderstand. I hope this improves the readability for the audience.

Specific pointers are outlined below. Apologies for any repetition with earlier comments—these examples illustrate some (though not all) of the areas where such issues were observed.

Abstract – there are now over 277K structural models

This number has now been updated.

Page 3 second paragraph
“The PDB offers precomputed sequence clusters at certain thresholds, including 100%, 95%, 50%, and 30%, based on the MMseqs2 algorithm”

– the PDB is referred to but the PDB itself is just the archive, it would be more accurate to refer to which website is being used (one or all of RCSB PDB, PDBE or PDBj)

We have corrected this and have made additional changes throughout the manuscript to reflect referring to the correct website. We have also included a paragraph at the beginning discussing

“Many of these selections can be made using the PDB API32, which allows for automated filtering of relevant structural data.”
Here, it would be relevant to be specific that this API is from RCSB PDB or make it more general and cite the APIs of both PDBe and PDBj
It might be worth a sentence in the intro to disambiguate the wwPDB from the different sites

We have now cited APIs form PDBe, PDBj, and RSCB.

Page 6
“For X-ray crystallography structures, resolution is determined by the diffraction limit of Bragg reflections, defined by the maximum scattering angle at which measurable reflections are observed”
Please add a reference, although, does the audience know what a Bragg reflection is?
To refine the scope of this paper, we have elected to remove the following sentences:

‘For X-ray crystallography structures, resolution is determined by the diffraction limit of Bragg reflections, defined by the maximum scattering angle at which measurable reflections are observed. Meanwhile, for cryo-EM, Fourier Shell Correlation (FSC) at 0.143 is the standard criterion for estimating resolution[^35^](https://paperpile.com/c/ORduAs/z64g).’

“Exploring these metrics can be important”
Strengthen this sentence to be “Exploring these metrics is important”

We have strengthened this sentence, which now states: ‘As structural models can vary widely in quality, these validation metrics are important to consider to maintain scientific reliability and to minimize the risk of errors propagating into biological interpretation, drug design, or computational modeling.’

“The evaluation of experimental data includes checking for twinning in X-ray crystallography data41 or completeness of chemical shift data in NMR42 “
I’m not sure this benefits the audience unless you go into what twinning is or why someone would care – I see this as more data quality and here you want to talk about model fit to data. They are subtly different and for this audience, I would avoid data quality discussions or you will need to greatly expand the discussion on it to make it meaningful.

We agree and have removed these sentences in our effort to refine the piece for our intended audience.

Random note: why are Q-scores not discussed?

This is a great point. We have added information on Q-scores and other metrics used with cryo-EM structures. The paragraph is below:

In cryo-EM, there is ongoing development of robust validation metrics, with an expanding set of approaches being introduced to evaluate the quality of deposited maps and models[^48^](https://paperpile.com/c/ORduAs/b3bQ). Because density maps are reconstructed from noisy, heterogeneous particle populations, they frequently exhibit substantial variation in local resolution. Global measures such as Fourier Shell Correlation (FSC) between half-maps remain the gold standard for estimating overall map resolution, while model–map FSC curves assess the consistency between the atomic model and experimental density[^49,50^](https://paperpile.com/c/ORduAs/VfD9+EuF5). Increasingly, local validation has become critical. EMRinger evaluates the accuracy of side-chain placement by comparing density peaks with expected rotamer positions, and Q-scores quantify how well the density supports individual atoms and residues[^51,52^](https://paperpile.com/c/ORduAs/7oyP+VoZB).

Page 7 paragraph 2 – It is detailed that all these metrics are important to consider but no help is given in knowing what a good model is – either detail this or link to where the reader could discover more. It may be worth referring to the model quality sliders, which are uniform across the PDB member websites or the validation cif and documentation.
This whole section is too light and short for what is needed, the reader is left knowing something has to be reviewed – which is good and the general direction of the assement but ultimately still in the dark as to what to look for.

No mention of Molprobity
Could also mention these <https://www.ebi.ac.uk/pdbe/api/doc/validation.html>
And also the validation reports

We currently have metrics, including MolProbity and PDB validation information, in Recommendation #2. We added the following sentence on how people should use these metrics when comparing structures.

When comparing two groups of structures, it is crucial to balance confounding variables to ensure that biological differences, rather than methodological or crystallographic artifacts, drive the observed differences. Differences in resolution, space group, unit‐cell parameters, data processing, and data collection parameters can lead to false positives or negatives. Depending on the question, this can also include differences in local metrics such as MolProbity or validation scores.

Recommendation 3
I strongly disagree that the level of reader who is reading this guide would be able to 1) reprocess data and 2) make a better job of it than PDBredo.
The space used for recommendation 3 would be better used to expand recommendation 2.

As this recommendation talks about both other reprocessing databases (PDB-REDO) and refers to other articles that can help users get started with refinement if they want to, we feel as though it fits within the scope of this piece. Even if users may not be re-refining, it is important them to understand the potential implications of this choice.

Rec 4
Expand on anisotropic B-factors – you do expand on B factors later but that needs to be here

We have added the following sentence to better describe anisotropic B-factors and how best to use them.

‘These include anisotropic B-factors, alternative atom locations (altlocs), or multiple models. These features reflect how structural data capture atomic heterogeneity. Anisotropic B-factors describe the direction and magnitude of atomic displacement, while alternative atom locations (altlocs) represent multiple conformations modeled for a single atom. Multiple models, often used in ensemble structures, provide different plausible conformations that together capture the underlying structural variability.’

Recommendation 5
This section is also a list of things to do, but with little help to the reader how to do them or where to find out about them. Who is this guide for?

We believe this fits within the scope of the paper as we expect this will be for anyone who might be interested in structural bioinformatics including people who have some background in statistics or others who may be more novice. However, we did add in some examples and sentences to provide context of the statistical tests.

‘When comparing two unpaired groups, choose parametric or non-parametric tests based on data distribution. Parametric tests assume normality, while non-parametric tests are more flexible when distributions are skewed (e.g., residue-level B-factor values or pocket volumes).’

Recommendation #6:
“Comparing structures with very similar crystallographic parameters” what does this mean exactly? What is ‘very similar’?
“ensuring similar crystallographic properties such as resolution, space group, and unit cells.” This isn’t correct, While it’s true that comparing structures from the same crystal form (e.g., same space group and unit cell) can help isolate biological effects, such matches are rare and not always necessary. Tools like structural alignment can handle different space groups and cell parameters effectively.
The whole paragraph between pages 8-9 needs more thought, it is not clearly written and this is making it misleading. This is the weakest area of the manuscript as it is quiet muddled, it needs to re-thought conceptually.

We have clarified this point, empahsizing what we mean by ‘Comparing structures with very similar crystallographic parameters’.

‘We recommend pairing structures based on biological differences and ensuring that they have similar crystallographic properties. Some general guidelines include using datasets with resolutions within 0.3 Å, identical space groups, and unit cell dimensions within 10% of each other. While these criteria are not always achievable, deviations can introduce artifacts: differences in crystal contacts or solvent volume may affect the conclusions you can draw.’

“If the PDBs come from the same protein” this is a weird turn of phase and a bit science colloquial

We agree and have clarified this statement.

‘Comparing structures of the same protein, you can compare using chain and residue IDs, but a standard numbering scheme is required.’

“you can compare using chain and residue IDs, but a standard numbering scheme is required. This can be done by manually renumbering chains and residues or employing algorithms such as PDBRenum to map PDB residue numbers onto UniProt numbering, which will also allow integration with other databases (see Recommendation #9)75. If PDBs are related, you can also align them based on a multiple sequence alignment.”
You mention SIFTS before (and then in the next section) but not here, why? Maybe this all should be brough together.
It would also be good to mention the PDBe-KB protein pages for structural comparison and clustering
<https://www.ebi.ac.uk/pdbe/pdbe-kb/>
<https://www.ebi.ac.uk/pdbe/pdbe-kb/proteins/2etx>
<https://pubmed.ncbi.nlm.nih.gov/34755867/>

Thank you for these points. We have added and referenced these points, including bringing SIFT into this section.

In section 9, page 10
Would be worth mentioning the PDBe ligand pages
<https://www.ebi.ac.uk/pdbe-srv/pdbechem/chemicalCompound/show/STI>
<https://pubmed.ncbi.nlm.nih.gov/40100137/>
And PDBe CCDUtils
<https://pubmed.ncbi.nlm.nih.gov/38042830/>

Thank you for these references. We have now included them.

“However, users must remain mindful of the “last-Ångstrom” problem, “
What does this mean exactly? It is not explained anywhere and is not a common phrase

We had added an explanation about this phenomenom.

Reviewer #2: The author gives a short review-style overview of how to deal with structure models from the PDB. Although it is not a unique overview (there have for instance been many book chapters on the topic), it is a good read for anyone who is relatively new to structural bioinformatics. It is easy to read and covers all the big issues in dealing with PDB entries. I do have some notes that may improve the manuscript:
• Introduction, first paragraph: Please rephrase the first sentence. Helen Berman has done a lot for the PDB over many years, but she was not in the lead when the PDB was founded.

Thank you, we have reflected this in our opening sentence.

• Introduction, first paragraph: The PDB does not contain structures but models thereof. Please use ‘structure models’ or ‘entries’.

‘In 1971, with only seven structures, the first, and still active, open-access database in biology was created: the Protein Data Bank (PDB)[^1^](https://paperpile.com/c/ORduAs/sSRz). From these modest beginnings, the PDB has grown to include over 277,000 structures[^2^](https://paperpile.com/c/ORduAs/KVx2), and its systematic archiving of macromolecular models has reshaped the field, transforming our understanding of the relationship between structure and biological function and enabling advances from elucidating enzyme catalysis to the rational design of new therapeutics.’

Agree. This is now reflected in the manuscript.

• Introduction, first paragraph: The PDB is not only robust, it was also the first (still active) open-access resource in biology.

Thank you. We have included this information in the opening paragraph of the manuscript.

‘In 1971, with only seven structures, the first, and still active, open-access database in biology was created: the Protein Data Bank (PDB)[^1^](https://paperpile.com/c/ORduAs/sSRz). From these modest beginnings, the PDB has grown to include over 277,000 structures[^2^](https://paperpile.com/c/ORduAs/KVx2), and its systematic archiving of macromolecular models has reshaped the field, transforming our understanding of the relationship between structure and biological function and enabling advances from elucidating enzyme catalysis to the rational design of new therapeutics.’

• Page 3, first line: Please elaborate a bit on the symmetry-related protomers. These can be purely coincidental with non-crystallographic symmetry, part of a homo-multimer, or part of a homomultimer that also needs application of crystallographic symmetry. Explain that you can download biological assemblies from the PDB.

We have included information on this in recommendation #4 (The PDB and structural models are weird and biased.)

• P3, end of Recommendation #1: The mention API is not the PDB API, but rather the RCSB-API. Please also mention that PDBe has a very rich API. I don’t know about PDBj, but the author should look into that.

Thank you for pointing out this oversight. We have included information to point to all PDB APIs.

• P3, last paragraph: NMR entries do not a have a reported resolution.

Thank you for pointing out this oversight. We have removed that.

• P4, 2nd paragraph: The PDB doesn’t mandate anything in terms of models having a minimal quality. It just test for the quality with is validation pipeline and make those results public. You can essentially still deposit rubbish. Please refer to the papers from the different validation taskforces from the wwPDB such as Read, et al. (see: <https://www.wwpdb.org/task/validation-task-forces>).

Thank you. We have corrected this language.

• P4, 2nd paragraph: AFAIK the twinning evaluation in the PDB validation server is done by Xtriage which should probably be cited. Density validation things originally all came from the Electron Density Server (Kleywegt et al., 2004). Currently the RS is calculated by the program density-fitness (van Beusekom et al, 2018). See <https://www.wwpdb.org/validation/XrayValidationReportHelp>

We have updated these references.

• P5, first paragraph: Please also mention DNATCO which is a validation to for DNA/RNA backbones.

We have included a reference to DNATCO.

For nucleic acids, PDB-REDO has introduced validation routines to assess the normality of Watson–Crick base-pair geometry, while DNATCO provides complementary validation of DNA and RNA backbone conformations.

• Recommendation #3: I think it is written as ‘PDB-REDO’ nowadays.

Thank you. We have updated the manuscript to reflect this.

• Recommendation #3: MTZ is a data format. The recommendation in 1998 was about deposition of reflection data, not about the format. Please also note that deposition of reflection data became mandatory in 2008. There are roughly 10k crystallographic PDB entries without reflection data.

Thank you for pointing this out. We have updated the language to reflect this.

• P6, 2nd paragraph: The team behind PDB-REDO published a paper about backfilling missing loops by homology with a program called Loopwhole. Please have a look at that.

Thank you for pointing this out. We have added a sentence referencing this paper.

‘The PDBRedo team has developed an algorithm (Loopwhole) to help fill in many of these missing loops, but it is most effective when high-quality homologous structures are available and when the experimental electron density supports accurate grafting and refinement. Models that include filled loops will be classified as ‘rebuilt’ in the PDBRedo database.’

• Page 6, end of Recommendation #4: I couldn’t agree more!

Thank you!

• P7, 2nd par: there is a reference to #5 in #5.

That was added by accident and has been removed.

• Recommendation #9: Please mention the PDBe-KB which is an important resource in this context. Perhaps also mention the 3D-Beacons network that connects many structural biology resources.

We had added this important resource to recommendation #9.

• P11, first paragraph: In the context of AlphaFold and missing ligands and co-factors, the author could point to AlphaFill which tries to fill this void.

Thank you for this suggestion. We have added this reference and citation.

Reviewer #3: This is a useful opinion piece on how to conduct structural bioinformatics studies. It follows a long series of "Ten Simple Rules" pieces published in PLOS CB. It is well written and quite clear. It provides suitable guidance for people new to structural bioinformatics techniques.

I just have a few things that could be added, expanded, or emphasized.

1) The distinction between asymmetric units and biological assemblies should be described. The PDB provides separate mmCIF and PDB-format files for biological assemblies. Usually these come from the authors, but some of them are also generated by software (PISA). The latter occurs when the PISA assembly is larger than the authors' biological assembly. For most scientific studies, only the biological assemblies should be used. The ms could warn readers that about 20% of the assemblies in the PDB are incorrect. The ProtCID and ProtCAD databases are useful sources for sorting out true from false assemblies.

We have included information on this in recommendation #4 (**The PDB and structural models are weird and biased.)**

2) In a similar vein, the author describes the importance of crystal parameters in separating structures. This is the idea of "crystal forms" used in our ProtCID and ProtCAD databases. The idea is that to understand protein assemblies, it is the number of unique crystal forms that is truly informative (e.g. on identifying the correct homodimer for a set of homologous family of proteins). This is also applicable for studying loop conformations that may be in crystal contacts. Understanding and grouping a set of structures into crystal forms can allow someone to analyze loop conformations in different crystallographic contexts in order to eliminate the confounding role of crystallization artifacts.

We agree and have added a paragraph in recommendation #8 to reflect this.

In some cases, it is valuable to collect structures with diverse crystallographic properties from the same or closely related proteins. Such comparisons can provide insight into conformational heterogeneity and, in particular, are useful for studying loop conformations that crystal contacts may influence. By grouping structures into distinct crystal forms, one can analyze loop conformations across different crystallographic contexts and disentangle genuine biological flexibility from artifacts introduced during crystallization.

3) I think a few words are in order for the efforts of Helen Berman and colleagues in remediating the PDB data after they took over from Brookhaven in 1998. These efforts are the absolute foundation of our being able to perform structural bioinformatics. There are several papers from Berman et al that could be cited. The effort is ongoing and includes efforts by PDBe and PDBj.

Thank you for pointing out this missing piece of information. We have added in the following paragraph:

‘Critically, when the Research Collaboratory for Structural Bioinformatics (RCSB) PDB, one of their first major undertakings was a large-scale remediation of legacy data. Early entries suffered from inconsistent formats, incomplete metadata, and non-standard nomenclature, making systematic analysis nearly impossible. The remediation effort, later extended by the wwPDB partners (PDBe, PDBj, and BMRB), standardized chemical components, corrected errors, and transitioned the archive to the more robust mmCIF format[^2–4^](https://paperpile.com/c/ORduAs/KVx2+rNsT+YxSJ). These efforts laid the foundation for structural bioinformatics by ensuring that PDB data were reliable, interoperable, and machine-readable.’

4) I think there could be a short section on the value of the mmCIF format. It is much richer than PDB format. It is necessary for structures with new ligands, which now have 5 characters and therefore are not available in legacy PDB format. Neither are large structures. An imporant feature of mmCIF is that it contains a mapping of the protein sequence to the residue numbering in the coordinates. That is, one could number a protein chain from 1 to the length of the chain. But authors usually use a different numbering (e.g. from the Uniprot sequence). Different tables in the mmCIF files use either the author numbering or the 1-to-N numbering or both. Our PDBrenum database renumbers the author numbering in all tables so it agrees with the Uniprot numbers (it will be updated very soon and should be up-to-date going forward).

We agree and have inserted a paragraph reflecting this in the introduction along with a brief introduction of the information in the atom table.

5) We find the ECOD database to be very useful. for instance in comparing assignments to AFDB structures, TED/CATH is missing many domains altogether, while ECOD has most of them. Same for Zheng et al(2017) on p. 4.

This is a great point and have included it in the manuscript and a sentence reminding users that every database has its holes.

6) very minor: p.9: the SIFTS reference is given as "Velankar et al(2013)" instead of being a numbered reference.

Thank you. We have now updated this.

7) very minor: p.2: "whether your protein are part of large complexes" --> "whether your protein is part of large complexes"

Thank you. We have made this change.
