# Supplementary material for: Ten rules for a structural bioinformatic analysis
Source: PLoS Comput Biol. 2025 Oct 31;21(10):e1013094. doi: 10.1371/journal.pcbi.1013094 (PMC12578330; doi:10.1371/journal.pcbi.1013094)
Supplement: Attachment — Submitted filename: PLoS reviewer comments_104.docx [file pcbi.1013094.s002.docx]

- The number of released PDB entries is just over 242k, please correct the number in the manuscript. Not sure where the 277k came from, this might reflect the number of entries including things that are not released yet.

This has been corrected based on the PDB statistics on 10/4/2025 (<https://www.rcsb.org/stats/growth/growth-released-structures>).

- Please rephrase the sentence "By democratizing...novel discoveries" in the abstract.

We have rephrase the sentence as follows:

Through the democratization of structural data and open-source analytical tools, now amplified by the power of large language models, a broader community of researchers is equipped to drive new scientific discoveries using structural data.

- EM maps show electron potential rather than density. This should be corrected in several spots in the manuscript, e.g. by just using 'map' instead of 'density'.

We have corrected this wording throughout the manuscript.

- Recommendation 2: NMR entries do not have a reported resolution.

We have clarified that the section discussing resolution does not reflect NMR structures.

Additional factors include resolution (not applicable for NMR), agreement with structure determination data, and stereochemical accuracy.

- Page 4, last sentence of first paragraph: It is also important to note that in boTh X-ray and cryo-EM, while two structures (or rather their underlying data) can have the same resolution, they can be modeled to different levels of ACCURACY, .....

We have updated the following sentence:

It is also important to note that in both X-ray and cryo-EM, while two structures can have the same resolution, they can be modeled to different levels of accuracy, necessitating the exploration of other metrics.

- Page 6, first paragraph: the statement of missing loops is somewhat in conflict with what the author says about Loopwhole in #2. Perhaps 'correct them' -> 'correct all of them'?

We have updated the sentence to reflect this.

Further, regions of unmodeled residues can arise for many reasons, including resolution and subjective modeling, but automated refinement pipelines cannot correct all of them.

- No need to add it at this stage, but on page 8 it would have been good to mention alignment free structure comparison. This can be done in torsion space (or an encoding thereof) or by using local distances such as with ProSMART or hydrogen bonds as done by HODER.

We have added a sentence on alignment-free structure comparisons.

Torsion-angle–based approaches preserve subtle, biologically meaningful differences that are often obscured in atomic coordinate space[^94,95^](https://paperpile.com/c/ORduAs/Cg1i+eUY0).
